# Genome sequence of *Epibacterium ulvae* strain DSM 24752^T^, an indigoidine-producing, macroalga-associated member of the marine *Roseobacter* group

**DOI:** 10.1186/s40793-019-0343-5

**Published:** 2019-08-06

**Authors:** Sven Breider, Shama Sehar, Martine Berger, Torsten Thomas, Thorsten Brinkhoff, Suhelen Egan

**Affiliations:** 1 Department of Biology of Geological Processes - Aquatic Microbial Ecology, Institute for Chemistry and Biology of the Marine Environment (ICBM), University of Oldenburg, Oldenburg, Germany; 2 Centre for Marine Science and Innovation (CMSI), School of Biological, Earth and Environmental Sciences, The University of New South Wales, Sydney, NSW 2052 Australia

**Keywords:** Biofilm, Indigoidine, Siderophore, *Roseobacter* group bacteria, α- proteobacteria

## Abstract

Strain U95^T^ (= DSM 24752^T^ = LMG 26464^T^) is the type strain of *Epibacterium ulvae*, which is the type species of the genus *Epibacterium*. This genus belongs to the marine *Roseobacter* group. *E. ulvae* Strain U95^T^ was isolated from the macroalga *Ulva australis*, is Gram-negative, rod-shaped and motile. Here we describe the permanent draft genome sequence and annotation of *E. ulvae* U95^T^ with a focus on secondary metabolite production and interaction with its host. The genome contains 4,092,893 bp, 3977 protein-coding genes and 60 RNA genes. The genome encodes a gene cluster for synthesis of the blue-pigmented secondary metabolite indigoidine and contains several genes for adhesion mechanisms, putative bacteriocin, siderophores, a type VI secretion system, and enzymes that confer oxidative stress resistance. Combined, these features may aid in the successful colonization and persistence of *E. ulvae* on host surfaces and in competition with the surrounding microbial consortium.

**Electronic supplementary material:**

The online version of this article (10.1186/s40793-019-0343-5) contains supplementary material, which is available to authorized users.

## Introduction

The genus *Epibacterium* was proposed by Penesyan et al., [41] and belongs to the family *Rhodobacteraceae* within the class *Alphaproteobacteria*. Currently, the genus comprises two closely related strains (U82 and U95^T^) which both belong to the species *Epibacterium ulvae*. The name refers to the source of isolation of the strains, the surface of the alga *Ulva australis*. The type strain *Epibacterium ulvae* U95^T^ is a motile, rod-shaped bacterium, which is oxidase and catalase positive and known to produce a so far uncharacterised antibacterial compound. *E. ulvae* belongs to the *Roseobacter* group which is ubiquitous in the marine environment and can account for up to 25% of the total marine bacterial community, e.g. in coastal sea-water [58]. To date, the majority of characterized *Roseobacter* strains have been isolated from seawater, however, an increasing number of *Roseobacter* group organisms are found in association with eukaryotic organisms, such as macroalgae [12, 44, 54], phytoplankton [4, 28] and marine invertebrates [6, 9, 15], where they can establish both beneficial [27, 57] and pathogenic relationships [30, 51].

The production of various secondary metabolites has been described for several *Rhodobacteraceae*, e.g. tropodithietic acid (TDA) [10], roseobacticides [52] and indigoidine [16, 20]. Several *Roseobacter* group members are photoheterotrophs using light to generate ATP via aerobic anoxygenic photosynthesis, have the ability to oxidise carbon monoxide [36, 56] or play an important role in the marine sulphur cycle [29].

Here we present the draft genome of the *E. ulvae* type strain U95^T^ focussing on genes involved in secondary metabolism and host association. The aim of this study is to aid the identification and characterization of potentially novel biologically active metabolites and to gain a deeper understanding of the adaptive features of this member of the *Roseobacter* group.

## Material and methods

### Growth conditions, genomic DNA isolation and phenotypic assays

**Fig. 1:**
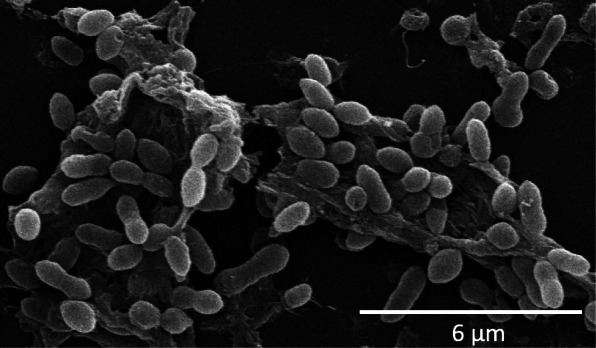
Scanning electron micrograph of *Epibacterium ulvae* U95^T^ showing the characteristic variations in cell morphology

*Epibacterium ulvae* U95^T^ was obtained from the surface of the marine alga *Ulva australis,* collected at Sharks Point, Clovelly, a rocky intertidal zone on the east coast of Sydney, Australia [41]. General features of *E. ulvae* U95^T^ are reported in Additional file 1: Table S1. *E. ulvae* U95^T^ was grown in Difco™ Marine Broth 2216 (MB) and incubated at 25 °C for approx. 18 h with agitation at 150 rpm on an orbital shaker (Ratek, Australia). Genomic DNA was extracted using the XS DNA extraction protocol [55]. Cell morphology was examined using scanning electron microscopy (Fig. 1). The ability of *E. ulvae* U95^T^ to form biofilms was performed using a 96 well polystyrene microtiter plate assay [33, 39]. Purification and analysis of indigoidine was examined using the previously described method by [16], but with Marine Broth agar plates instead of YTSS agar plates. The secretion of iron-scavenging siderophores was tested using the Chrome Azurol S (CAS) liquid assay [2, 49].

### Genome sequencing, assembly and annotation

The genome was sequenced on the Illumina sequencing platform both at the Joint Genome Institute and the Ramaciotti Centre for Genomics (UNSW). Reads were assembled using the Spades assembler [7] and the assembly was checked for potential contamination by blastn against the nucleotide (nt) database, respectively [3]. The genome assembly was annotated using the IMG platform [34]. All sequencing project information is reported in Additional file 1: Table S2. A phylogenetic tree of 16S rRNA gene sequences from *E. ulvae* U95^T^ and closely related strains was calculated using the Neighbour Joining method and the maximum likelihood method in ARB [32] with 1000 bootstrap replicates. Only sequences with more than 1265 bp were considered in the calculations.

## Results and discussion

*E. ulvae* U95^T^ was chosen for genome sequencing to aid in the characterization of potentially novel biologically active metabolites and to gain a deeper understanding of the adaptive features of this member of the *Roseobacter* group. The complete genome sequence has been deposited at NCBI (GenBank accession number PHJF00000000) and Integrated Microbial Genome (IMG) (Genome ID 2747842406).

The draft genome consists of 25 contigs containing 4,092,893 bp and a content of 52.95% G + C. Of the 4,037 total genes 3,977 are protein-coding genes and 60 are RNA genes (47 tRNA and 3 RNA genes, 10 other). 79.76% of the protein coding genes are assigned to functions, 18.75% remained as hypothetical proteins Table 1. COG functional categories are shown in Table 2. A pulsed-field gel electrophoresis showed, that U95^T^ contains at least eight plasmids [41], but this could not be assigned in the genome sequence due to its draft status.

**Table 1:** Genome statistics

Attribute	Value	% of Total
Genome size (bp)	4,092,893	100
DNA coding (bp)	3,665,857	89.57
DNA G+C (bp)	2,167,294	52.95
DNA scaffolds	25	
Total genes	4037	100
Protein coding genes	3977	98.51
RNA genes	60	1.49
Pseudo genes	0	0
Genes in internal clusters	830	20.56
Genes with function prediction	3220	79.76
Genes assigned to COGs	2805	69.48
Genes with Pfam domains	3308	81.94
Genes with signal peptides	324	8.03
Genes with transmembrane helices	819	20.29
CRISPR repeats	0	0

**Table 2:** Number of genes associated with general COG functional categories

Code	Value	%age	Description
J	190	6.09	Translation, ribosomal structure and biogenesis
A	n.a.	n.a.	RNA processing and modification
K	265	8.49	Transcription
L	99	3.17	Replication, recombination and repair
B	2	0.06	Chromatin structure and dynamics
D	29	0.93	Cell cycle control, Cell division, chromosome partitioning
V	48	1.54	Defense mechanisms
T	119	3.81	Signal transduction mechanisms
M	164	5.25	Cell wall/membrane biogenesis
N	55	1.76	Cell motility
U	74	2.37	Intracellular trafficking and secretion
O	140	4.48	Posttranslational modification, protein turnover, chaperones
C	190	6.09	Energy production and conversion
G	233	7.46	Carbohydrate transport and metabolism
E	375	12.01	Amino acid transport and metabolism
F	82	2.63	Nucleotide transport and metabolism
H	167	5.35	Coenzyme transport and metabolism
I	127	4.07	Lipid transport and metabolism
P	184	5.89	Inorganic ion transport and metabolism
Q	88	2.82	Secondary metabolites biosynthesis, transport and catabolism
R	282	9.03	General function prediction only
S	176	5.64	Function unknown
-	1232	30.52	Not in COGs

The total is based on the total number of protein coding genes in the genome

The total does not correspond to 3977 CDS coding DNA sequence because some genes are associated with more than one COG functional categories

**Fig. 2:**
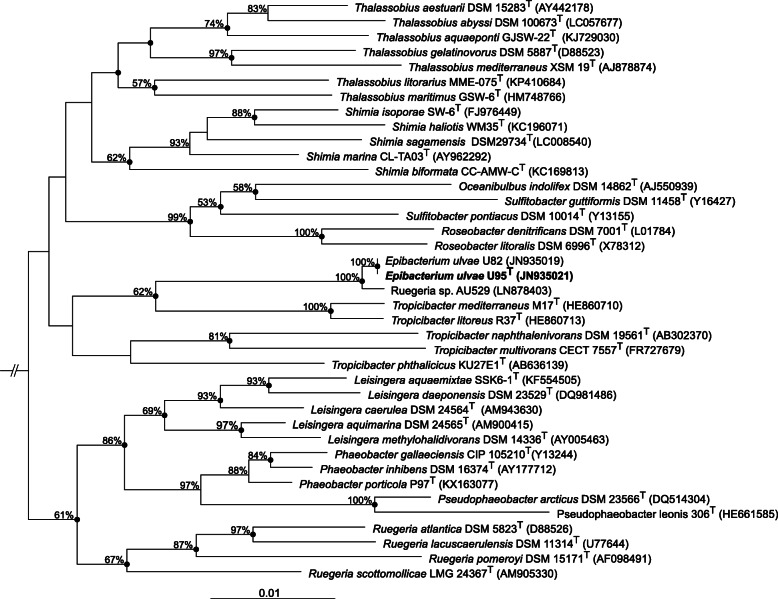
Phylogenetic tree highlighting the position of *Epibacterium ulvae* U95^T^ relative to the type species of the neighboring genera *Tropicibacter*, *Thalassobius, Sulfitobacter* and *Shimia*. The tree was calculated using the Neighbour Joining method in ARB [32] with 1000 bootstrap replicates; only values > 50% are shown at major nodes. Filled circles indicate nodes also recovered reproducibly with the maximum likelihood method. Bar equals 0.01 substitutions per nucleotide position. Only sequences with more than 1265 bp were considered in the calculations

On the basis of 16S rRNA gene sequence analysis, the genus *Epibacterium* represents a separate branch within the class *Alphaproteobacteria*. A blastn search of the 16S rRNA gene against the GenBank database revealed that U95^T^ is closely related to members of the genera *Tropicibacter*, *Thalassobius*, *Sulfitobacter* and *Shimia*. A phylogenetic tree calculated using the Neighbour Joining method and the Maximum Likelihood method supported this finding. Interestingly, *Tropicibacter litoreus* and *Tropicibacter mediterraneus* cluster together with the genus *Epibacterium* and not with other members of the genus *Tropicibacter* (Fig. 2). The blastn search also revealed that U95^T^ is 99% identical over 1324 bp (99% total length) to strain *Ruegeria* sp. AU529 (Acc # LN878403) which also clusters with *Epibacterium ulvae* U82 and U95^T^. Strain AU529 was isolated from the marine sponge *Cymbastela concentrica*, sampled in Botany Bay, NSW, Australia [21], which is in close distance to Sharks Point from where U95^T^ was isolated.

### Genomic features related to pigment and siderophore production

**Fig. 3:**
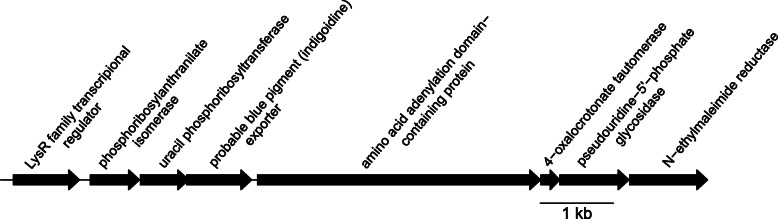
Indigoidine gene cluster in *Epibacterium ulvae* U95^T^. The genes shown are located at the locus tags Ga0207336_104202 - Ga0207336_104209. Additional information regarding gene homology to other indigoidine producing organisms can be found in Additional file 1: Table S3

Genome analysis of *E. ulvae* U95^T^ revealed a cluster of genes responsible for the production of indigoidine. Indigoidine (5,5′-diamino-4,4′-dihydroxy-3,3′- diazadiphenoquinone-(2,2′) is a natural, water-insoluble blue pigment synthesized through the condensation of two units of L-glutamine by a 4′-phosphopantetheinyl transferase (PPTase) non-ribosomal peptide synthases (NRPS) module. Homologs of this gene are found in a number of environmental bacteria for example, IndC/ IdgC from *Dickeya dadantii* (*Erwinia chrysanthemi*) [13], IndC from *Streptomyces aureofaciens* CCM 3239 [37], IbpA from *Streptomyces lavendulae* [40] and IgiD from *Rhodobacterales* sp. Y4I (previously *Phaeobacter* sp., Y41) [16]. Indigoidine is considered as a powerful radical scavenger enabling phytopathogens to combat oxidative stress, organic peroxides and superoxides during the plant defence response [46]. The blue pigment has also been shown to possess antimicrobial activity [16]. Chemical extraction of *E. ulvae* U95^T^ resulted in a blue colored solution. Subsequent High-Pressure Liquid Chromatography / Mass Spectrometry (HPLC/MS) analysis identified peaks corresponding to indigoidine (Additional file 1: Figure S3). U95^T^ was previously shown to have antimicrobial activity through production of a yet uncharacterized compound [42]. Using a heterologous gene expression approach an *E. coli* clone containing a fosmid with a genomic insert from strain U95^T^ was shown to harbour the indigoidine gene cluster (fosmid 19F10, GenBank accession number JX523956). This clone also displayed potent antibacterial and antinematodal (*Caenorhabditis elegans*) activity [42]. Our current analysis of the genome and the pigment suggests that this antimicrobial activity may be attributed to indigoidine. Based on comparative analysis to the indigoidine gene clusters from other bacteria (Additional file 1: Table S3) we propose that the putative indigoidine gene cluster of U95^T^ includes the genes Ga0207336_104203 to Ga0207336_104208 (Fig. 3).

The genome of *E. ulvae* U95^T^ also encodes a cluster for biosynthesis and transport of a putative iron-chelating siderophore (Ga0207336_10111, Ga0207336_10110, Ga0207336_1019, Ga0207336_1018, Ga0207336_1017and Ga0207336_10120 – Ga0207336_10117, respectively). Siderophores are small organic molecules (approximately 200–2000 Da) produced by some microorganisms under iron-limiting conditions that in turn sequester iron from different sources [48]. Iron (Fe) is an essential element for growth of almost all living microorganisms. It plays an important role in several metabolic processes, including the tricarboxylic acid cycle, electron transport chain, oxidative phosphorylation and detoxification of reactive oxygen species (e.g., catalases, super-oxidase dismutase) [1]. The organisation of the biosynthesis gene cluster in U95^T^ is identical to the siderophore gene cluster found in *Phaeobacter inhibens* DSM 17395 and 2.10, which is hypothesized to be petrobactin-like [54]. Sterile filtrated supernatant from culture of *E. ulvae* U95^T^ growing under iron depleted conditions showed CAS activity, indicating the excretion of siderophores. The negative control, *Roseobacter denitrificans* DSM 7001, which lacks the gene cluster, showed no evidence of siderophore production (Additional file 1: Figure S1).

**Fig. 4:**
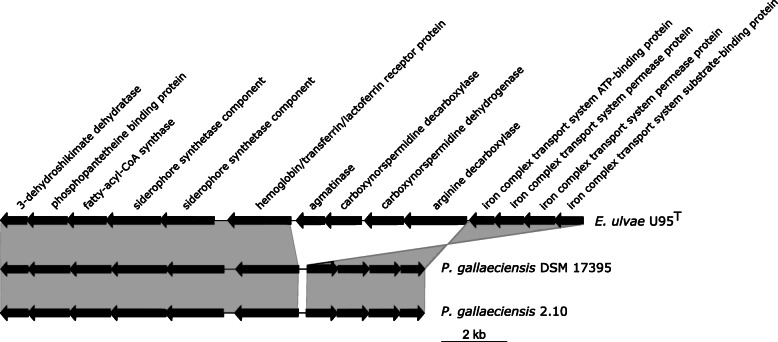
Gene cluster for siderophore production and transport in *Epibacterium ulvae* U95^T^, *Phaeobacter gallaeciensis* DSM 17395 and *Phaeobacter gallaeciensis* 2.10. Grey boxes show conserved gene orders. Note that the genes for the iron complex transport system are inverted in *Phaeobacter* spp. The genes shown are located on the locus tags (from left to right) Ga0207336_1017 - Ga0207336_10124 in *E. ulvae* U95^T^

*E. ulvae* U95^T^ contains four additional genes within the siderophore cluster that are absent in other *Phaeobacter* spp. but are found in some roseobacters isolated from the accessory nidamental gland of *Euprymna scolopes* [14] (Fig. 4). These additional four genes, encoding an arginine decarboxylase, a carboxynorspermidine dehydrogenase, a carboxynorspermidine decarboxylase and an agmatinase likely encode for the production of spermidine. Thus, these proteins could be involved in the synthesis of the polyamine backbone of the siderophore. Polyamines such as putrescine, spermidine and norspermidine are the backbone of siderophores putrebactin, petrobactin [8] and vulnibactin [38] respectively.

### Genomic features related to surface colonization and biofilm formation

Chemotaxis can be used by motile bacterial cells, e.g. to sense and swim towards metabolites suitable for growth or to avoid toxic substances. The ability for an organism to use chemotaxis can provide a competitive advantage in natural environment [19, 61]. *E. ulvae* U95^T^ has genes for several chemotaxis receptors as well as signal transducers (Additional file 1: Table S4), suggesting that this strain is responsive to a wide variety of stimuli, including, for example, nutrients, growth conditions, changes in osmolarity, quorum signals, and antibiotics.

The genome of *E. ulvae* U95^T^ encodes for several extracellular components and surface appendages likely involved in surface colonization and biofilm formation. These include genes encoding for flagellar assembly, surface lipoproteins, capsular polysaccharide (O-antigen) and Flp pilli (Additional file 1: Table S5). In most natural environments, biofilms are the prevailing lifestyle of bacteria. Bacterial biofilms consist of cells adhered to a solid surface and encased in an extracellular polymeric matrix [24]. To effectively attach and colonise a surface, bacteria make use of different adhesion factors including surface proteins [47], curli, fimbriae, pili [17, 35] and flagella [60] in addition to the production of extracellular polymeric substances (EPS). We assessed the ability of *E. ulvae* U95^T^ to form biofilms using a 96 well polystyrene microtiter plate assay [33, 39]. These biofilm formation experiments revealed that *E. ulvae* is able to form a dense biofilm on polystyrene and after 72 h it was as effective in biofilm formation as other tested strains of the *Roseobacter* group (Additional file 1: Figure S2).

### Genomic features related to stress response and defence mechanism

All bacteria have an inherent tendency to sense and respond to changing environmental conditions. The genome of U95^T^ encodes several multidrug efflux pumps and drug resistance proteins (Additional file 1: Table S6) as well as permeases of the drug/ metabolite transporter (DMT) superfamily (Additional file 1: Table S7) that can provide additional protective benefit to U95^T^ against antibiotics and toxins secreted by competing microorganism. U95^T^ was shown to be resistant to the aminoglycoside antibiotics gentamicin and spectinomycin [41]. We found genes belonging to the aminoglycoside-phosphotransferase family (Locus: Ga0207336_105156; Ga0207336_110112, Predicted kinase, aminoglycoside phosphotransferase (APT) family; ATP-dependent O-phosphorylation by phosphotransferases (APH); pfam01636), which are known to be responsible for resistance to aminoglycosides in other bacteria and likely to provide U95^T^ with resistance to these antibiotics.

Macroalgae and other photosynthetic organisms use reactive oxygen species (ROS) as a means to protect themselves against incoming colonizing microorganisms or pathogenic attacks [59]. Genome analysis of *E. ulvae* U95^T^ revealed the presence of genes for a catalase-peroxidase (Ga0207336_104247) and a superoxide dismutase (Ga0207336_102167, Ga0207336_101691) that may provide a defence mechanism against the oxidising environment associated with the macroalgal surface.

Interestingly, the genome also encodes for several genes with homology to those involved in detoxification, including an uncharacterised protein associated with tellurium resistance (YaaN, Ga0207336_102395), quaternary ammonium compound-resistance protein (SugE, Ga0207336_104105), and czcA/czcB (Ga0207336_11334, Ga0207336_11335) for resistance to cobalt, zinc and cadmium [5, 25, 43, 53]. While the precise role for these genes in *E. ulvae* U95^T^ is not known, heavy metal resistance has been described to occur in some sponge associated bacteria [50] as well as in bacteria isolated from marine habitats [18]. Therefore, the presence of these genes in U95^T^ might provide the bacterium with protection against various contaminations and xenobiotics found in the marine ecosystem, which can be particularly high in urbanised coastal regions [11].

### Genomic features related to sulfur cycling

The genome of *E. ulvae* U95^T^ contains genes for a dimethylsulfoxide (DMSO) reductase (Ga0207336_10987), a periplasmic dimethylsulfoxide reductase YedY (Ga0207336_105258) and a trimethylamine-N-oxide (TMAO) reductase YedZ (Ga0207336_105259), suggesting this bacterium may play a role in dimethylsulfoniopropionate (DMSP) degradation and DMSO conversion. Bacteria of the *Roseobacter* group are generally involved in the transformation of DMSP by demethylation or via the cleavage pathway [31] and thus play an important role in the marine sulphur cycle.

### Genomic features related to protein secretion systems

Protein secretion systems play a major role in modulating various bacterial interactions (biofilms and mutualistic or pathogenic associations) within their environments. The genome of U95^T^encodes for the full set of genes for a type III secretion system (T3SS), type IV secretion system (T4SS) and type VI secretion system (T6SS) (Additional file 1: Table S8). T4SSs can be found in almost all bacterial species where they play an important role in the transportation of monomeric and multimeric proteins (toxins and nucleoprotein complexes) as well as DNA across the bacterial cell envelope [45]. The T3SS and T6SS secretion mechanisms are thought to play an important role in both pathogenic and bacterial-host symbioses where they function to transport a variety of substrates (proteins, toxins and enzymes) directly into host cells via a needle-like structure ([26, 62]). The potential for *E. ulvae* U95^T^ to utilize a variety of secretion mechanisms may thus represent an important mechanism of host adaptation in this epiphytic bacterium.

## Conclusions

The genome of *E. ulvae* U95^T^ revealed several characteristics that are potentially beneficial for its persistence and survival on host surfaces. As discussed above several adhesins including curli, fimbrae and pilli may allow this bacterium to interact with a variety of hosts including algae and sponges. Genes encoding for production of toxins, bacteriocins, antimicrobials, capsule polysaccharides and siderophores were identified and these could provide additional protective benefit towards competing microbiota in environmentally stressful conditions. This study represents the first genome description of a member of the *Epibacterium* genus and may guide future research aimed at characterizing the role of specific genes and pathways involved marine host-microbe interactions.

## Additional file

Additional file 1: **Table S1** Classification and general features of *Epibacterium ulvae* U95 T in accordance with the MIGS recommendations [22] published by the Genome Standards Consortium [23]. **Table S2** Project information. **Table S3** Gene comparisons of putative indigoidine biosynthesis clusters. **Table S4** Gene clusters encoding proteins for chemotaxis-related methyl accepting proteins *E. ulvae* U95 T. **Table S5** Gene clusters encoding proteins and extracellular s tructures for surface in *E. ulvae* U95 T. **Table S6** Resistance proteins and transporters present in genome of *Epibacterium ulvae* U95 that provide protection against competing microorganisms. Drug resistance proteins and efflux pumps. **Table S7**. Resistance proteins and transporters present in genome of *Epibacterium ulvae* U95 that provide protection against competing microorganisms. DMT Transporter. **Table S8** Protein secretory systems present in Epibacterium ulvae U95. **Figure S1.** Chrome Azurol S (CAS) assay for the determination of siderophore production. The strains were grown on iron depleted medium and the sterile filtrated supernatant of *E. ulvae* U95 T and *R. denitrificans* (R.d.) were used for the CAS assay. Deferoxamine mesylate (50 μM) used as positive control (+) and the iron depleted medium as negative control (−). **Figure S2.** Biofilm forming ability of different isolates under study. Error bars represents standard deviations from multiple cultures (*n* = 10). **Figure S3.** Above: PDA-chromatogram at 610 nm; below: MS mass chromatogram at m/z 249. (PDF 1182 kb)

## Abbreviations

CAS: Chrome Azurol S
DMSO: dimethylsulfoxide
DMSP: dimethylsulfoniopropionate
EPS: extracellular polymeric substances
IMG: Integrated Microbial Genome
MB: Marine Broth 2216
ROS: reactive oxygen species
T3SS: type III secretion system
T4SS: type IV secretion system
T6SS: type VI secretion system
TDA: tropodithietic acid
