# Supplementary material for: Genome sequence of Epibacterium ulvae strain DSM 24752T, an indigoidine-producing, macroalga-associated member of the marine Roseobacter group
Source: Environ Microbiome. 2019 Aug 6;14:4. doi: 10.1186/s40793-019-0343-5 (PMC7989816; doi:10.1186/s40793-019-0343-5)
Supplement: Supplementary file 1 — Table S1 Classification and general features of Epibacterium ulvae U95 T in accordance with the MIGS recommendations [22] published by the Genome Standards Consortium [23]. Table S2 Project information. Table S3 Gene comparisons of putative indigoidine biosynthesis clusters. Table S4 Gene clusters encoding proteins for chemotaxis-related methyl accepting proteins E. ulvae U95 T. Table S5 Gene clusters encoding proteins and extracellular s tructures for surface in E. ulvae U95 T. Table S6 Resistance proteins and transporters present in genome of Epibacterium ulvae U95 that provide protection against competing microorganisms. Drug resistance proteins and efflux pumps. Table S7. Resistance proteins and transporters present in genome of Epibacterium ulvae U95 that provide protection against competing microorganisms. DMT Transporter. Table S8 Protein secretory systems present in Epibacterium ulvae U95. Figure S1. Chrome Azurol S (CAS) assay for the determination of siderophore production. The strains were grown on iron depleted medium and the sterile filtrated supernatant of E. ulvae U95 T and R. denitrificans (R.d.) were used for the CAS assay. Deferoxamine mesylate (50 μM) used as positive control (+) and the iron depleted medium as negative control (−). Figure S2. Biofilm forming ability of different isolates under study. Error bars represents standard deviations from multiple cultures (n = 10). Figure S3. Above: PDA-chromatogram at 610 nm; below: MS mass chromatogram at m/z 249. (PDF 1182 kb) [file 40793_2019_343_MOESM1_ESM.pdf]

Supplementary Information for:

Genome sequence of *Epibacterium ulvae* strain DSM 24752^T, an indigoidine producing surface associated member of the marine Roseobacter clade

Authors: Sven Breider[1#], Shama Sehar[2#], Torsten Thomas[2], Thorsten Brinkhoff[1] and Suhelen Egan[2*]

Postal address:

[1]Department of Biology of Geological Processes - Aquatic Microbial Ecology, Institute for Chemistry and Biology of the Marine Environment (ICBM), University of Oldenburg, Oldenburg, Germany

[2]Centre for Marine Bio-Innovation, School of Biological, Earth and Environmental Sciences, The University of New South Wales, Sydney, NSW, Australia

[*]Corresponding author: s.egan@unsw.edu.au

# contributed equally to the study

Table S1. Classification and general features of *Epibacterium ulvae* U95^T in accordance with the MIGS recommendations (Field et al., 2008) published by the Genome Standards Consortium (Field et al., 2011).

MIGS ID	Property	Term	Evidence code[a]
	Classification	Domain *Bacteria*	TAS (Woese et al., 1990)
		Phylum *Proteobacteria*	TAS (Garrity et al., 2005)
		Class *Alphaproteobacteria*	TAS (Garrity et al., 2005)
		Order *Rhodobacterales*	TAS (Garrity et al., 2005)
		Family *Rhodobacteraceae*	TAS (Garrity et al., 2005)
		Genus *Epibacterium*	TAS (Penesyan et al., 2013)
		Species *Epibacterium ulvae*	TAS (Penesyan et al., 2013)
		(Type) strain: U95^T (DSM 24752^T)	TAS (Penesyan et al., 2013)
	Gram stain	negative	TAS (Penesyan et al., 2013)
	Cell shape	Rod-shaped	TAS (Penesyan et al., 2013)
	Motility	Motile	TAS (Penesyan et al., 2013)
	Sporulation	not reported	NAS
	Temperature range	12 to 34 °C	TAS (Penesyan et al., 2013)
	Optimum temperature	24 to 26 °C	TAS (Penesyan et al., 2013)
	pH range; Optimum	6–9; 7-8	TAS (Penesyan et al., 2013)
	Carbon source	Amino acids, oligosaccharides, sugar alcohols	TAS (Penesyan et al., 2013)
MIGS-6	Habitat	Marine	TAS (Penesyan et al., 2013)
MIGS-6.3	Salinity	1 to 6% NaCl (w/v)	TAS (Penesyan et al., 2013)
MIGS-22	Oxygen requirement	Aerobic	TAS (Penesyan et al., 2013)
MIGS-15	Biotic relationship	unknown	NAS
MIGS-14	Pathogenicity	not reported	NAS
MIGS-4	Geographic location	Sydney, Australia	TAS (Penesyan et al., 2013)
MIGS-5	Sample collection	April 2007	NAS
MIGS-4.1	Latitude	33° 51′ 09″ S	TAS (Penesyan et al., 2013)
MIGS-4.2	Longitude	151° 16′ 00″ E	TAS (Penesyan et al., 2013)
MIGS-4.4	Altitude	Not reported	NAS

[a] Evidence codes - IDA: Inferred from Direct Assay; TAS: Traceable Author Statement (i.e., a direct report exists in the literature); NAS: Non-traceable Author Statement (i.e., not directly observed for the living, isolated sample, but based on a generally accepted property for the species, or anecdotal evidence). These evidence codes are from the Gene Ontology project (Ashburner et al., 2000).

Table S2. Project information.

MIGS ID	Property	Term
MIGS 31	Finishing quality	Standard Draft
MIGS-28	Libraries used	Nextera
MIGS 29	Sequencing platforms	Illumina HiSeq 2500-1TB
MIGS 31.2	Fold coverage	130x
MIGS 30	Assemblers	Spades 3.10.1
MIGS 32	Gene calling method	IMG Annotation Pipeline v.4.15.1
	Locus Tag	
	GenBank ID	PHJF00000000
	GenBank Date of Release	21th November 2017
	GOLD ID	
	BIOPROJECT	
MIGS 13	Source Material Identifier	SAMN04488118
	Project relevance	Biodiscovery

Table S3. Gene comparisons of putative indigoidine biosynthesis clusters

E. ulvae U95[T] IMG locus tag	IMG Annotation	% Amino acid identity to homolog in *Rhodobacterales* sp. Y41[a]	% Amino acid identity to homolog in *Dickeya dadantii*[a]	% Amino acid identity to homolog in *Streptomycetes lavendulae* [a]
Ga0207336_ 104203	Phosphoribosylanthranilate isomerase	31.8[b] (EDZ46365)	NA	66.5 (BAW81998) *orf3*
Ga0207336_ 104204	Uracil phosphoribosyltransferase	NA	30.2 (ABM97471)	56.5 (BAW81997) *orf4*
Ga0207336_ 104205	Probable blue pigment (indigoidine) exporter	NA	48 (P42194) *pecM*	58.1 (BAW81996) *orf5*
Ga0207336_ 104206	Indigoidine synthease	42 (EDZ47671) *igiD*	44 (ADN00615) *indC/idgC*	45.5 (BAW81991) *ibpA*
Ga0207336_ 104207	4-oxalocrotonate tautomerase	39.2 (EDZ45410) *igiF*	NA	NA
Ga0207336_ 104208	Pseudouridine-5'-phosphate glycosidase	43.3[b] (EDZ48351)	56.9 (ADN00617) *indA/ idgA*	59.2 (BAW81994) *orf7*
Ga0207336_ 104209	N-ethylmaleimide reductase	43.6 [b] (EDZ47493)	38.3[b] (ADM99362)	58.6 (BAW81993) *orf8*

[a] Gene information from IMG and GenBank databases with reference to genes described in Cude et. al. 2012 for *Rhodobacterales* sp. Y41, Chu et. al. 2010, Glasner et. al. 2011 for *Dickeya dadantii* and Pait et. al. 2017 for *Streptomycetes lavendulae*. GenBank accession number in brackets. NA, not applicable, no homolog of the gene in the respective organism. [b] Homolog found but not located near the proposed indigodine cluster.

References:

Ashburner, M., Ball, C.A., Blake, J.A., Botstein, D., Butler, H., Cherry, J.M., Davis, A.P., Dolinski, K., Dwight, S.S., Eppig, J.T, Harris, M.A., Hill, D.P., Issel-Tarver, L., Kasarskis, A., Lewis, S., Matese, J.C., Richardson, J.E., Ringwald, M., Rubin, G.M., Sherlock, G. 2000. Gene ontology: tool for the unification of biology. The Gene Ontology Consortium. Nat.Genet. 25 (1) 25–29.

Chu, M. K., Lin, L. F., Twu, C. S., Lin, R. H., Lin, Y. C., Hsu, S. T., Tzeng, K. C., Huang, H. C. 2010. Unique features of *Erwinia chrysanthemi* (*Dickeya dadantii*) RA3B genes involved in the blue indigoidine production. Microbiol Res 165: 483-495

Cude, W.N., Mooney, J., Tavanaei, A. A., Hadden, M.K., Frank, A.M., Gulvik, C.A., May, A.L., Buchan, A., 2012. Production of the antimicrobial secondary metabolite indigoidine contributes to competitive surface colonization by the marine Roseobacter *Phaeobacter* sp. strainY4I. Appl. Environ. Microbiol. 78 (14), 4771–4780.

Field D, Amaral-Zettler L, Cochrane G, Cole JR, Dawyndt P, Garrity GM, Gilbert J, Glöckner FO, Hirschman L, Karsch-Mzrachi I, Hans-Peter, K., Knight, R., Kottmann, R., Kyrpides, N., Meyer, F., Gil, I.S., Sansone, S.A., Schriml, L.M., Sterk, P.,Tatusova, T., Ussery, D.W., White, O., Wooley, J., 2011. The Genomic Standards Consortium. PLoS Biol. 9: e1001088.

Field, D., Garrity, G., Gray, T., Morrison, N., Selengut, J., Sterk, P., Tatusova, T., Thomson, N., et al. 2008. The minimum information about a genome sequence (MIGS) specification. Nat. Biotechnol.;26:541-547.

Garrity, G.M., Bell, J.A., Lilburn, T., Phylum, XIV. *Proteobacteria* phyl. nov. *In*: Brenner DJ, Krieg NR, Staley JT, Garrity GM, editors. Bergey's Manual of Systematic Bacteriology, second edition, vol. 2 (The Proteobacteria), part B (The Gammaproteobacteria), Springer, New York, 2005, p. 1.

Glasner, J. D., et al. (35 co-authors) 2011 Genome sequence of the plant-pathogenic bacterium *Dickeya dadantii* 3937." J. Bacteriol 193: 2076-2077.

Pait, I. G. U., Kitani, S., Kurniawan, Y. N., Asa, M., Iwai, T., Ikeda, H., Nihira, T. 2017. Identification and characterization of lbpA, an indigoidine biosynthetic gene in the gamma-butyrolactone signaling system of *Streptomyces lavendulae* FRI-5 J Biosci Bioeng 124: 369-375

Penesyan, A., Breider, S., Schumann, P., Tindall, B.J., Egan, S., Brinkhoff, T., 2013 *Epibacterium ulvae* gen. nov, sp. nov., epibiotic bacteria isolated from the surface of a marine alga. Int. J. Syst. Evol. Microbiol. 63:1589–1596.

Woese, C.R., Kandler, O., Wheelis, M.L., 1990. Towards a natural system of organisms: proposal for the domains *Archaea, Bacteria*, and *Eucarya.* Proc. Natl. Acad. Sci. USA. 87:4576-4579.

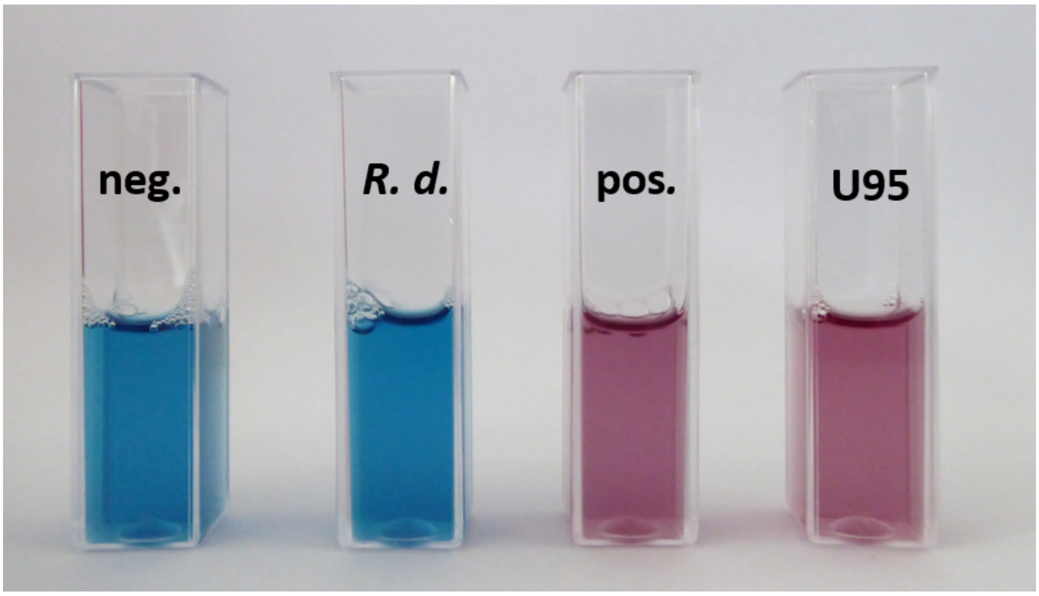

Figure S1: Chrome Azurol S (CAS) assay for the determination of siderophore production. The strains were grown on iron depleted medium and the sterile filtrated supernatant of *E. ulvae* U95^T and *R. denitrificans* (R.d.) were used for the CAS assay. Deferoxamine mesylate (50µM) used as positive control (+) and the iron depleted medium as negative control (-).

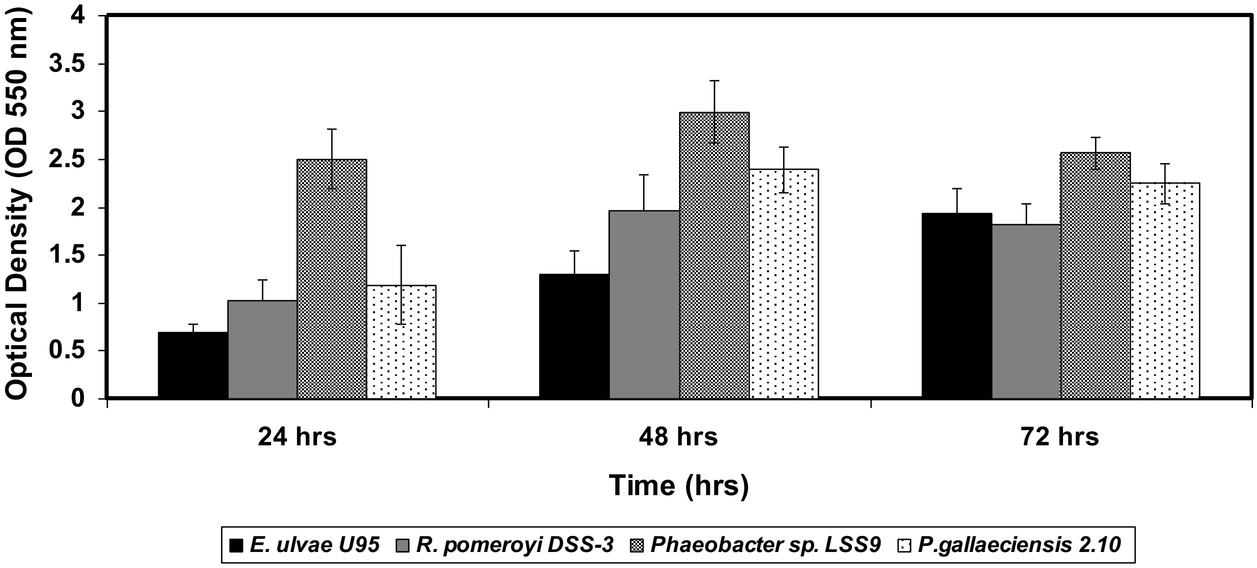

Figure S2: Biofilm forming ability of different isolates under study. Error bars represents standard deviations from multiple cultures (n = 10).

Table S4. Gene clusters encoding proteins for chemotaxis-related methyl accepting proteins *E. ulvae* U95^T.

Gene ID	Locus Tag	IMG Product Name
2747873791	Ga0207336_103427	CheB methylesterase
2747873790	Ga0207336_103426	CheD activator of MCP protein methylation
2747873609	Ga0207336_103245	chemotaxis protein methyltransferase CheR
2747875493	Ga0207336_1128	chemotaxis protein MotA
2747875515	Ga0207336_11230	chemotaxis protein MotB
2747873349	Ga0207336_102607	chemotaxis protein MotB
2747874552	Ga0207336_10658	methyl-accepting chemotaxis protein
2747875423	Ga0207336_11165	methyl-accepting chemotaxis protein
2747873839	Ga0207336_103475	methyl-accepting chemotaxis protein
2747874548	Ga0207336_10654	methyl-accepting chemotaxis protein
2747875083	Ga0207336_10913	methyl-accepting chemotaxis protein
2747873773	Ga0207336_103409	methyl-accepting chemotaxis protein
2747872551	Ga0207336_101599	methyl-accepting chemotaxis sensory transducer
2747872375	Ga0207336_101423	methyl-accepting chemotaxis protein
2747874858	Ga0207336_107156	methyl-accepting chemotaxis sensory transducer with Cache sensor
2747874519	Ga0207336_10625	methyl-accepting chemotaxis sensory transducer with Pas/Pac sensor
2747873608	Ga0207336_103244	CheW protein
2747873607	Ga0207336_103243	two-component system chemotaxis sensor kinase CheA

Table footer

Table S5. Gene clusters encoding proteins and extracellular structures for surface in *E. ulvae* U95^T.

Gene ID	Locus Tag	IMG Product Name
2747874132	Ga0207336_104281	Flp pilus assembly protein TadD
2747874855	Ga0207336_107153	Flp pilus assembly protein TadD
2747872038	Ga0207336_10185	Flp pilus assembly protein TadG
2747872040	Ga0207336_10187	Flp pilus assembly protein TadG
2747874847	Ga0207336_107145	pilus assembly protein CpaB
2747874134	Ga0207336_104283	pilus assembly protein CpaC
2747874848	Ga0207336_107146	pilus assembly protein CpaC
2747874851	Ga0207336_107149	pilus assembly protein CpaF
2747874853	Ga0207336_107151	type II secretion system protein F (GspF)
2747874852	Ga0207336_107150	type II secretion system protein F (GspF)

Table footer

Table S6. Resistance proteins and transporters present in genome of *Epibacterium ulvae* U95 that provide protection against competing microorganisms. Drug resistance proteins and efflux pumps.

Gene ID	Locus Tag	IMG Product Name
2747873967	Ga0207336_104116	osmoprotectant transport system substrate-binding protein
2747873673	Ga0207336_103309	multidrug efflux pump subunit AcrB
2747872907	Ga0207336_102163	multidrug efflux pump
2747872733	Ga0207336_101781	multidrug efflux pump subunit AcrB
2747873067	Ga0207336_102324	MATE family multidrug resistance protein
2747872998	Ga0207336_102255	MATE family multidrug resistance protein
2747874230	Ga0207336_104379	antitoxin YefM
2747871959	Ga0207336_1016	ATP-binding cassette subfamily B multidrug efflux pump
2747873956	Ga0207336_104105	quaternary ammonium compound-resistance protein SugE
2747872748	Ga0207336_1024	predicted MFS family arabinose efflux permease
2747872642	Ga0207336_101690	predicted MFS family arabinose efflux permease
2747874733	Ga0207336_10730	response regulator receiver modulated diguanylate cyclase

2747872693	Ga0207336_101741	stress-induced morphogen
2747874663	Ga0207336_106169	two-component system response regulator TctD
2747875528	Ga0207336_11243	response regulator receiver protein
2747873033	Ga0207336_102290	uncharacterized tellurite resistance protein B-like protein
2747873034	Ga0207336_102291	uncharacterized tellurite resistance protein B-like protein
2747873049	Ga0207336_102306	broad specificity phosphatase PhoE
2747874902	Ga0207336_1084	Ca2+-binding RTX toxin-like protein
2747875860	Ga0207336_11626	camphor resistance protein CrcB
2747874440	Ga0207336_105205	chloramphenicol-sensitive protein RarD
2747875426	Ga0207336_11169	choline dehydrogenase
2747873843	Ga0207336_103479	invasion protein IalB
2747872617	Ga0207336_101665	invasion protein IalB
2747873656	Ga0207336_103292	MerR family redox-sensitive transcriptional activator SoxR
2747873101	Ga0207336_102358	DHA1 family bicyclomycin/chloramphenicol resistance-like MFS transporter
2747872527	Ga0207336_101575	DHA1 family bicyclomycin/chloramphenicol resistance-like MFS transporter
2747873982	Ga0207336_104131	DHA1 family bicyclomycin/chloramphenicol resistance-like MFS transporter
2747873181	Ga0207336_102438	DHA1 family tetracycline resistance protein-like MFS transporter

Table S7. Resistance proteins and transporters present in genome of *Epibacterium ulvae* U95 that provide protection against competing microorganisms. DMT Transporter.

Gene ID	Locus Tag	IMG Product Name
2747874805	Ga0207336_107103	drug/metabolite transporter (DMT)-like permease
2747875154	Ga0207336_10984	drug/metabolite transporter (DMT)-like permease
2747872338	Ga0207336_101386	drug/metabolite transporter (DMT)-like permease
2747874242	Ga0207336_1056	drug/metabolite transporter (DMT)-like permease
2747874804	Ga0207336_107102	drug/metabolite transporter (DMT)-like permease

Table footer

Table S8: Protein secretory systems present in *Epibacterium ulvae* U95.

COG ID	Gene ID	IMG Product Name	Locus Tag	Category	Similarity to	Homology (% ID/Protein coverage)
COG4965	2747874852	type II secretion system protein F (GspF)	Ga0207336_107150	Type II	*Leisingera caerulea* DSM 24564	81/100
COG2064	2747874853	type II secretion system protein F (GspF)	Ga0207336_107151	Type II	*Nautella italica* CECT 7645	75/97
COG4789	2747874136	type III secretion protein V	Ga0207336_104285	Type III	*Chelativorans* sp. BNC1	57/98
COG4669	2747874141	type III secretion protein J	Ga0207336_104290	Type III	Rhodobacteraceae bacterium (ex *Bugula neritina* AB1)	49/84
COG1157	2747874144	type III secretion system FliI/YscN family ATPase	Ga0207336_104293	Type III	Rhodobacteraceae bacterium (ex *Bugula neritina* AB1)	67/99
-	2747874147	type III flagellar switch regulator (C-ring) FliN	Ga0207336_104296	Type III	Burkholderia sp. ABCPW 14	39/68
COG4790	2747874148	type III secretion protein R	Ga0207336_104297	Type III	Rhodobacteraceae bacterium (ex *Bugula neritina AB1*)	68/98
COG4794	2747874149	type III secretion protein S	Ga0207336_104298	Type III	Rhodobacteraceae bacterium (ex Bugula neritina AB1)	66/96

COG4791	2747874150	type III secretion protein T	Ga0207336_104299	Type III	Rhodobacteraceae bacterium (ex Bugula neritina AB1)	50/98
COG1377	2747874151	flagellar biosynthetic protein FlhB/type III secretion protein U	Ga0207336_104300	Type III	Rhodobacteraceae bacterium (ex Bugula neritina AB1)	45/95
COG3505	2747872854	type IV secretion system protein VirD4	Ga0207336_102110	Type IV	*Roseobacter* sp. Gal101 Conjugal transf prot TraG	63/93
COG3505	2623166881	type IV secretion system protein VirD4	Ga0207336_11539	Type IV	*Sulfitobacter pseudonitzschiae* DSM 26824 conjugal transfer protein TraG	94/100
COG0630	2747875803	type IV secretion system protein VirB11	Ga0207336_11540	Type IV	*Sulfitobacter pseudonitzschiae* DSM 26824	90/99
COG2948	2747875804	type IV secretion system protein VirB10	Ga0207336_11541	Type IV	*Sulfitobacter pseudonitzschiae* DSM 26824	84/100
COG3504	2747875805	type IV secretion system protein VirB9	Ga0207336_11542	Type IV	*Sulfitobacter pseudonitzschiae* DSM 26824	89/100
COG3736	2747875806	type IV secretion system protein VirB8	Ga0207336_11543	Type IV	*Sulfitobacter pseudonitzschiae* DSM 26824	59/99
-	2747875808	type IV secretion system protein VirB6	Ga0207336_11545	Type IV	*Sulfitobacter pseudonitzschiae* DSM 26824	65/99

-	2747875810	type IV secretion system protein VirB5	Ga0207336_11547	Type IV	*Sulfitobacter pseudonitzschiae* DSM 26824	79/99
COG3451	2747875811	type IV secretion system protein VirB4	Ga0207336_11548	Type IV	*Sulfitobacter pseudonitzschiae* DSM 26824	92/100
COG3702	2747875812	type IV secretion system protein VirB3	Ga0207336_11549	Type IV	*Sulfitobacter pseudonitzschiae* DSM 26824	88/100
-	2747875813	type IV secretion system protein VirB2	Ga0207336_11550	Type IV	*Sulfitobacter pseudonitzschiae* DSM 26824	80/100
COG3501	2747874076	type VI secretion system secreted protein VgrG	Ga0207336_104225	Type VI	*Tropicibacter multivorans* CECT 7557	75/97
COG4104	2747874078	uncharacterized Zn-binding protein involved in type VI secretion	Ga0207336_104227	Type VI	*Tropicibacter multivorans* CECT 7557	86/100
COG3515	2747874082	type VI secretion system protein ImpA	Ga0207336_104231	Type VI	*Tropicibacter multivorans* CECT 7557	68/88
-	2747874083	type VI secretion system secreted protein Hcp	Ga0207336_104232	Type VI	*Tropicibacter multivorans* CECT 7557	85/100
COG3516	2747874084	type VI secretion system protein ImpB	Ga0207336_104233	Type VI	*Tropicibacter multivorans* CECT 7557	92/100
COG3517	2747874085	type VI secretion system protein ImpC	Ga0207336_104234	Type VI	*Tropicibacter multivorans* CECT 7557	

COG3517	2747874086	type VI secretion system protein ImpD	Ga0207336_104235	Type VI	*Tropicibacter multivorans* CECT 7557	90/97
COG3518	2747874087	predicted component of type VI protein secretion system	Ga0207336_104236	Type VI	*Tropicibacter multivorans* CECT 7557	61/99
COG3519	2747874088	type VI secretion system VasI/ImpG family protein	Ga0207336_104237	Type VI	*Tropicibacter multivorans* CECT 7557	71/100
-	2747874089	type VI secretion system (T6SS) VasB/ImpH family protein	Ga0207336_104238	Type VI	*Tropicibacter multivorans* CECT 7557	55/100
COG0542	2747874090	type VI secretion system protein VasG	Ga0207336_104239	Type VI	*Tropicibacter multivorans* CECT 7557	86/100
COG3521	2747874093	type VI secretion system VasD/TssJ family lipoprotein	Ga0207336_104242	Type VI	*Tropicibacter multivorans* CECT 7557	72/83
COG3522	2747874094	type VI secretion system protein ImpJ	Ga0207336_104243	Type VI	*Tropicibacter multivorans* CECT 7557	76/100
COG3455	2747874095	type IV / VI secretion system ImpK/NasF family protein	Ga0207336_104244	Type VI	*Tropicibacter multivorans* CECT 7557	68/97
COG3523	2747874096	type VI protein secretion system component VasK	Ga0207336_104245	Type VI	*Tropicibacter multivorans* CECT 7557	66/98

-	2747872209	sec-independent protein translocase protein TatA	Ga0207336_101257	Tat	*Sedimentitale nanhaiensis* DSM 24252	83/98
COG1826	2747872210	sec-independent protein translocase protein TatB	Ga0207336_101258	Tat	*Phaeobacter gallaeciensis* DSM 26640	73/89
COG0805	2747872211	sec-independent protein translocase protein TatC	Ga0207336_101259	Tat	*Nautella italica* LMG 24365	88/100
COG0653	27478775357	Protein translocase subunit secA	Ga0207336_110147	Sec	*Phaeobacter gallaeciensis* DSM 26640	85/99
COG1952	2747875546	Protein translocase subunit secB	Ga0207336_11261	Sec	*Leisingera aquimarina* DSM 24565	84/94
COG0341	2747872895	protein translocase subunit secF	Ga0207336_102151	Sec	*Tritonibacter horizontis* O3.65	81/100
COG0342	2747872896	protein translocase subunit secD	Ga0207336_102152	Sec	*Leisingera aquamixtae* SSK6-1	86/100
COG1862	2747872897	protein translocase subunit yajC	Ga0207336_102153	Sec	*Salipiger mucosus* DSM 16094	
COG0706	2747875876	protein translocase subunit yidC	Ga0207336_11642	Sec	*Roseobacter* sp. SK209-2-6	76/100
COG0201	2747875850	protein translocase subunit secY/sec61 alpha	Ga0207336_11616	Sec	*Leisingera carulea* DSM 24564	93/100

COG0690	2747874526	protein translocase subunit secE/sec61 gamma	Ga0207336_10632	Sec	*Leisingera aquimarina* DSM 24565	94/100
COG1314	2747873230	protein translocase subunit secG	Ga0207336_102488	Sec	*Leisingera methylohalidivorans* DSM 14336	79/100

Table footer

LC-MS measurement of Indigoidin

Indigoidin

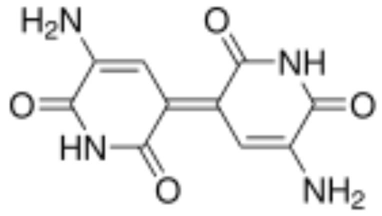

M	$C_{10}H_8N_4O_4$	molare Masse :	248,19	
M	$C_{10}H_8N_4O_4$	exakte Masse :	248.0546 Da	
$[M+H]^+$	$C_{10}H_9N_4O_4$	exakte Masse :	249.0624 Da	positive ESI-MS
$[M-H]^-$	$C_{10}H_7N_4O_4$	exakte Masse :	247.0467 Da	negative ESI-MS

HPLC conditions:

Column:
NUCLEODUR C18 Pyramid, 3 µm; length: 125 mm, ID 3 mm (Fa. Macherey-Nagel)

HPLC: Waters Alliance 2695
Eluent A: H_2O (0.05% HCOOH); Eluent B: MeOH (0.05% HCOOH)

Waters Alliance 2695 HPLC Pump Initial Conditions:

A%	95.0 Water
B%	5.0 ACN or MEOH

Flow (ml/min)	0.050
Stop Time (mins)	25.0
Column Temperature (°C)	25.0
Column Temperature Limit (°C)	5.0
Min Pressure (Bar)	0.0
Max Pressure (Bar)	345.0

Waters Alliance 2695 HPLC Pump Gradient Timetable
The gradient Timetable contains 7 entries which are :

Time	A%	B%	C%	D%	Flow	Curve
0.00	95.0	5.0	0.0	0.0	0.050	1
0.01	95.0	5.0	0.0	0.0	0.800	6
3.00	95.0	5.0	0.0	0.0	0.800	6
30.00	0.0	100.0	0.0	0.0	0.800	6
45.00	0.0	100.0	0.0	0.0	0.800	6
50.00	95.0	5.0	0.0	0.0	0.800	6
55.00	95.0	5.0	0.0	0.0	0.800	6

PDA: Waters996 PDA

Start Wavelength (nm)	200.00
End Wavelength (nm)	650.00

MS: „Q-TOF Micro“ Hybridmassspectrometer (Fa. Micromass)

Ionisation: Positive Elektrospray Ionisation “ESI+“

Chosen MS-Parameter:

Polarity	ES+
Capillary (V)	3200.0
Sample Cone (V)	35.0
Extraction Cone (V)	0.5
Desolvation Temp (°C)	250.0

Source Temp (°C)	100.0
Ion Energy (V)	2.5
Collision Energy	10.0
MCP Detector (V)	2300.0
TDC Start (mV)	900.0000
TDC Stop (mV)	100.0000

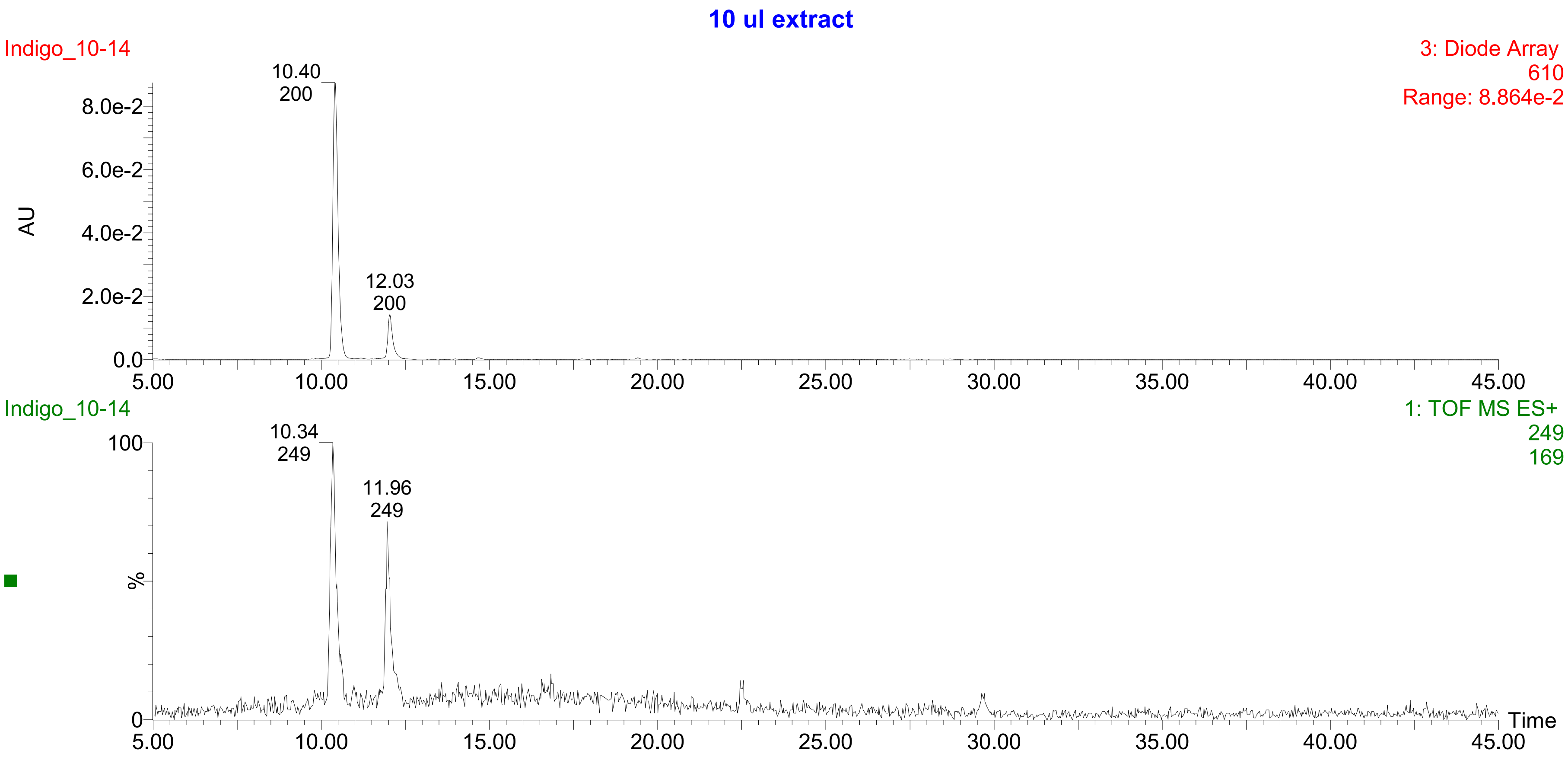

Figure S3: above: PDA-chromatogram at 610 nm; below: MS mass chromatogram at m/z 249.

Elemental composition Report for exact masses:

Peak at 10.40 min.:

Single Mass Analysis
Tolerance = 10.0 PPM / DBE: min = -1.5, max = 50.0
Selected filters: None
Monoisotopic Mass, Even Electron Ions
372 formula(e) evaluated with 3 results within limits (up to 50 closest results for each mass)

Mass	Calc. Mass	mDa	PPM	DBE	Formula	i-FIT	C	H	N	O
249.0623	249.0624	-0.1	-0.4	8.5	C10 H9 N4 O4	2773114.5	10	9	4	4
	249.0610	1.3	5.2	3.5	C9 H13 O8	2773176.3	9	13		8
	249.0637	-1.4	-5.6	13.5	C11 H5 N8	2773065.5	11	5	8	

Peak at 12.03 min.:

Single Mass Analysis
Tolerance = 20.0 PPM / DBE: min = -1.5, max = 50.0
Selected filters: None
Monoisotopic Mass, Even Electron Ions
372 formula(e) evaluated with 8 results within limits (up to 50 closest results for each mass)

Mass	Calc. Mass	mDa	PPM	DBE	Formula	i-FIT	C	H	N	O
249.0657	249.0664	-0.7	-2.8	12.5	C15 H9 N2 O2	35.2	15	9	2	2
	249.0669	-1.2	-4.8	5.5	H5 N14 O3	59.3		5	14	3
	249.0637	2.0	8.0	13.5	C11 H5 N8	39.8	11	5	8	
	249.0683	-2.6	-10.4	-0.5	C3 H13 N4 O9	53.7	3	13	4	9
	249.0624	3.3	13.2	8.5	C10 H9 N4 O4	39.9	10	9	4	4
	249.0696	-3.9	-15.7	4.5	C4 H9 N8 O5	49.7	4	9	8	5
	249.0704	-4.7	-18.9	16.5	C20 H9	35.9	20	9		
	249.0610	4.7	18.9	3.5	C9 H13 O8	41.4	9	13		8
